# Rearing of Black Soldier Fly Larvae with Corn Straw and the Assistance of Gut Microorganisms in Digesting Corn Straw

**DOI:** 10.3390/insects15100734

**Published:** 2024-09-24

**Authors:** Xifeng Wang, Xiangru Tian, Zhi Liu, Zhihua Liu, Shuying Shang, Haifeng Li, Jianhang Qu, Pengxiao Chen

**Affiliations:** 1 School of Biological Engineering, Henan University of Technology, Zhengzhou 450001, China; tianxr@stu.haut.edu.cn (X.T.); upzhil@stu.haut.edu.cn (Z.L.); 211060100222@stu.haut.edu.cn (Z.L.); 211170400523@stu.haut.edu.cn (S.S.); hfli@haut.edu.cn (H.L.); jhqu@haut.edu.cn (J.Q.); 2 School of International Education, Henan University of Technology, Zhengzhou 450001, China; 3 School of Food and Strategic Reserves, Henan University of Technology, Zhengzhou 450001, China; cpx2020@haut.edu.cn

**Keywords:** black soldier fly larvae (*Hermetia illucens)*, digestion, gut microbial community, cellulase-producing strain

## Abstract

**Simple Summary:**

The black soldier fly (*Hermetia illucens* L.) is a saprophagous insect belonging to the Stratiomyidae family that can degrade organic waste and convert it into high-quality insect protein and lipid. Corn straw is a kind of prevalent agricultural waste. In this study, black soldier fly larvae were reared with corn straw to obtain larval biomass and consume corn straw. The results showed that most of the black soldier fly larvae could survive and gain weight when fed with corn straw. Gut microorganisms could respond to the substrates, and four cellulase-producing strains isolated from the gut were beneficial to digesting corn straw.

**Abstract:**

Corn straw is considered a renewable biomass energy source, and its unreasonable disposal leads to resource waste and environmental pollution. Black soldier fly (*Hermetia illucens* L.) larvae (BSFL) facilitate the bioconversion of various types of organic wastes. In this study, we found that 88% of BSFL survived, and 37.4% of corn straw was digested after 14 days of feeding with corn straw. Contrary to expectations, the pretreatment of corn straw with alkaline hydrogen peroxide did not promote its digestion but rather reduced the growth and survival rates of BSFL. *Acinetobacter*, *Dysgonomonas*, and unclassified *Enterobacteriaceae* were the abundant genera in the BSFL gut fed with corn straw. Compared with the standard diet, the relative abundances of carbohydrate metabolism genes, such as the gene abundances of β-glucosidase and α-glucosidase, were higher with corn straw as the substrate. These results suggested that the gut microbial community could regulate suitable and functional microorganisms in response to the substrates. Furthermore, four cellulase-producing strains, namely *Klebsiella pneumoniae*, *Proteus mirabilis*, *Klebsiella oxytoca*, and *Providencia rettgeri*, were isolated from the guts of corn straw BSFL. These four strains helped increase the conversion rates of corn straw, the weights of BSFL, and survival rates. In summary, we reared BSFL with corn straw and discovered the functions of gut microorganisms in adapting to the substrates. We also isolated four cellulase-producing strains from the BSFL guts and declared the benefits of BSFL digesting corn straw.

## 1. Introduction

The annual agricultural crop residues, including straw from crops such as corn, wheat, and rice, amount to nearly 1 billion tons in China [1]. However, a large number of crop stalks are discarded or burned, bringing continuous environmental pollution, which is common in developing countries [2]. In order to avoid the waste of straw resources, animal feed, industrial use, and compost are the recommended methods, according to the Food Recovery Hierarchy [3]. Converting crop straw into valuable substances, such as crop straw-derived bioenergy and chemicals, has been a research focus for many years [4]. However, as a result of the recalcitrant structure of straw, the production of crop straw-derived bioenergy requires pretreatment using strong acid and alkali and the efficient enzymatic hydrolysis of straw [5]. Such requirements are not cost-effective and lead to secondary environmental pollution problems. In recent years, there has been growing interest in exploring alternative methods for converting straw into other valuable products.

Black soldier fly larvae (BSFL) are saprophytic insects that can quickly convert organic waste into insect protein and lipid compounds within their bodies [6]. BSFL biomass can be used as feedstuff for livestock, poultry, fish, and pets; thus, BSFL are insects with a remarkable industrialization prospect in the field of organic waste disposal [7,8]. The conversion of crop stalks by BSFL has attracted attention in recent years. Zheng et al. reported that after rearing BSFL with rice straw (30%) and restaurant solid waste (70%) for 10 days, about 65.5% of cellulose, 56.3% of hemicellulose, and 8.8% of lignin were digested [9]. Given the lack of continuous supply of restaurant solid waste, Manurung et al. attempted to rear BSFL with rice straw; however, they reported a waste reduction efficiency of only 10.85% [10]. To increase the digestion rate, Liu et al. applied alkaline peroxide pretreatment to change the structure of rice straw, remove part of the lignin from lignocellulose, and remove a little part of the hemicellulose. The conversion rate of rice straw was enhanced from 10.7% to 11.4% [11]. However, this conversion rate was still unremarkable. Actually, researchers have tried several other methods to feed BSFL with crop stalks. For example, Li et al. pretreated rice straw with 1% KOH, pretreated fermented rice straw with enzymes, and fed BSFL with the fermentation residues for biodiesel.

Querejeta et al. suggested that *Proteobacteria*, *Firmicutes*, and *Actinobacteriota* are the core microbiota among the BSF different developmental stages [12]. The gut microbial community in BSFL can also be shaped by feedstock and altered with the environment [13]. Liu et al. found that *Actinomyces*, *Dysgonomonas*, *Devosia,* and *Pelagibacterium* are the dominant flora when rice straw is the substrate [11]. Previous studies have shown that BSFL accelerate the degradation of kitchen waste, animal feces, antibiotics, aflatoxin B1, and some pesticides, with the gut microbial community playing a pivotal role in this process [14,15,16]. Meanwhile, *Bacillus velezensis* EEAM 10B isolated from BSFL guts could strengthen the nutrient metabolic process in BSFL via the changing gut microbiome and metabolic pathways, throwing light on the special contribution of some additional bacteria isolated from the gut in the rearing system [17]. Zhang et al. isolated several cellulose-degrading bacteria in BSFL guts and found that cellulose-degrading bacteria improved the conversion efficiency of the co-digestion of dairy and chicken manure by BSFL [18]. Whether the cellulose-degrading bacteria in BSFL guts can increase the conversion rate of crop straw remains unclear.

In this study, standard diet, corn straw, and alkaline hydrogen peroxide-pretreated corn straw were used as the substrates to feed the BSFL. The performances of the BSFL in these substrates were studied, including weight, survival rate, and digestion of corn straw. The community structure and composition of larval gut microbiota were analyzed. Four cellulase-producing bacteria were isolated from BSFL guts. These bacteria were added into the substrate digestion system, and the functions of these bacteria in corn straw digestion were studied.

## 2. Materials and Methods

### 2.1. Corn Straw Samples

The corn straw samples were obtained from the suburbs of the Henan Province. Initially, the samples were first cut into small pieces and then ground into particles (10–60 mesh) using a knife mill. The corn straw samples were pretreated as described by Liu et al. [11]. Briefly, 7.5 M NaOH solution was added to completely wet corn straw until the pH was 11.5, and 1 mL of 9.8 M H_2_O_2_ solution was added. The corn straw suspension was shaken at 150 rpm and 30 °C for 6 h, filtered, and washed with deionized water until it was neutral. Finally, the corn straw was frozen at −25 °C for 12 h and freeze-dried at −50 °C for 36 h. The standard diet was denoted as SD, which was composed of 75% wheat bran and 25% corn flour. The raw corn straw and alkaline hydrogen peroxide-pretreated corn straw samples were denoted as CS and PCS, respectively.

### 2.2. Rearing of BSFL

BSFL eggs were obtained from Henan University of Technology and fed with SD pre-soaked with 70% (*w*/*w*) water in a plastic box (28 cm × 18 cm × 18 cm). The plastic boxes were reared in an incubator at 70% humidity and 28 °C for 5 days [19]. Subsequently, 100 five-day-old larvae were selected for the bioconversion of the substrate and transferred to a plastic box (15 cm × 11 cm × 11 cm). Three substrates were applied in this study, including SD (standard diet, as control), CS (corn straw), and PCS (pretreated corn straw). Each substrate (15 g) was hydrated with approximately 100 mL of deionized water and incubated at 28 °C and 70% humidity for 24 h to ensure complete saturation before rearing the BSFL. Each substrate had three replicates, and all the substrates were placed in the boxes at once. The plastic boxes of BSFL were placed in a biochemical incubator at 28 °C and 70% humidity. The transfer time was labeled as day 0, and the weights of 10 randomly selected BSFL were recorded every 2 days and placed back in the plastic box. The live BSFL were counted every 2 days, and the dead BSFL were removed every day. After being reared for 16 days, the BSFL were picked out by a blunt tweezer (length 12.5 cm). The residues after the removal of the larvae were considered as frass. The frass of SD, CS, and PCS groups were labeled as SDL, CSL, and PCSL, respectively. The chemical compositions of the BSFL were analyzed as follows: The crude fat content of the BSFL was determined by an automatized Soxhlet extractor. Total ash was determined after mineralization at 550 °C for 3 h. The crude protein was determined with a Kjeldahl system.

### 2.3. Analysis of Corn Straw

The residual substrates SDL, CSL and PCSL were freeze-dried. The weights of the above three substrates were measured to determine the conversion rates. The conversion rate was calculated using the following equation: Conversion rate (%) = (weight of initial substrates−weight of residual substrates)/weight of initial substrates × 100. X-ray diffraction (XRD, D8ADVANCE, Bruker, Karlsruhe, Germany) was used to evaluate the crystallinity of the CS samples. The wavelength of the Cu radiation source was 1.5406 Å. The crystallinity index (CI_XRD_) of CS was calculated using the following equation: CI_XRD_ (%) = (I_002_ − I_am_)/I_002_ × 100, where I_002_ is the height of the crystalline peak at 22°, and I_am_ is the intensity of the peak at 18°. Scanning electron microscopy (SEM, QUANTA FEG 250, FEI, Hillsboro, OR, USA) was adopted to observe the surface morphology changes [11].

### 2.4. Gut DNA Extraction and Analysis

Ten larvae from each plastic box were randomly selected for the gut DNA extraction. Briefly, the larvae were disinfected in 75% alcohol for 3 min, washed with sterile water three times, and dissected with a scalpel on a dissecting pan. The midguts dissected in BSFL were immediately stored in an ultra-low temperature refrigerator at −80 °C for gut DNA extraction [20]. Total community genomic DNA extraction was performed using an E.Z.N.A™ (OMEGA Bio-Tek, Norcross, USA) Mag-Bind Soil DNA Kit (M5635-02, OMEGA Bio-Tek, Norcross, Georgia, USA), following the manufacturer’s instructions. The 16S rRNA V3–V4 amplicon was amplified using two primers, F (5′-CCTACGGGNGGCWGCAG-3′) and R (5′-GACTACHVGGGTATCTAATCC-3′). The reaction was set as follows: microbial DNA (10 ng/μL), 2 μL; forward primer (10 μM), 1 μL; reverse primer (10 μM), 1 μL; 2× Hieff Robust PCR Master Mix (10105ES03, Yeasen Biotechnology, Shanghai, China), 15 μL; water, 11 μL; and total 30 μL. The plate was sealed, and PCR was performed in a thermal instrument (Applied Biosystems 9700, Thermofisher, Waltham, Massachusetts, USA) using the following program: 1 cycle of denaturing at 95 °C for 3 min, first 5 cycles of denaturing at 95 °C for 30 s, annealing at 45 °C for 30 s, elongation at 72 °C for 30 s, 20 cycles of denaturing at 95 °C for 30 s, annealing at 55 °C for 30 s, elongation at 72 °C for 30 s, and a final extension at 72 °C for 5 min. The gut DNA samples were sequenced by Sangon Biotech, Shanghai, China, and bioinformatics analysis was performed as described [21]. Briefly, sequencing was performed using the Illumina MiSeq system (Illumina, San Diego, California, USA), according to the manufacturer’s instructions. After sequencing, sequences were quality filtered and denoised, and the chimera was removed using the DADA2 (Divisive Amplicon Denoising Algorithm 2) plugin. Then, the ASVs (amplicon sequence variants) were clustered by 100% similarity. Bacterial ASVs were classified taxonomically by blasting against the RDP Database. The taxonomy of the species annotation was divided into kingdom, phylum, class, order, family, and genus. All statistical analyses were performed with R statistical software (v3.6.0). Principal component analysis (PCA) was used to evaluate differences in the microbiome among the samples. The functional prediction analysis of bacteria using PICRUSt (v1.1.4) software was completed by comparing existing 16S rRNA gene sequencing data with a microbial reference genome database of known metabolic functions, enabling the prediction of bacterial metabolic functions.

### 2.5. Isolation and Identification of Cellulase-Producing Strains from the Gut

Ten larvae reared on CS were randomly selected for surface sterilization. Following aseptic treatment, the larvae were dissected, and their midguts were excised. The midgut was mixed with 10 mL of distilled water in a 50 mL flask and serially diluted up to 10^−5^. A 0.1 mL sample diluent was cultured in lysogeny broth solid medium (10 g/L tryptone, 5 g/L yeast extract, 10 g/L NaCl, and 10 g/L agar). Repeated plate streaking was applied to individual colonies until pure cultures were obtained. The pure cultures were cultured in CMC-Na medium (5 g/L CMC-Na, 0.5 g/L MgSO_4_, 2 g/L KH_2_PO_4_, 2.5 g/L peptone, 0.5 g/L yeast extract, and 15 g/L agar) and dyed with Congo red. The strains with the transparent zone were the cellulase-producing strains [22]. The cellulase activity of the strains was determined by CMC-Na and Whatman No. 1 filter paper, and the cellulase activities were set as CMC activity (CMCA) and filter paper activity (FPA). Briefly, the strains were cultured in lysogeny broth medium (10 g/L tryptone, 5 g/L yeast extract, and 10 g/L NaCl) at 30 °C with shaking at 160 rpm. Then, the cultures were centrifuged at 5000× *g* for 10 min to harvest the supernatant. The assay mixture consisted of 0.5 mL of supernatant, 1.5 mL of disodium hydrogen phosphate–citrate buffer, and either 1% (*w*/*v*) CMC-Na or the filter paper as a substrate. The mixture was incubated at 37 °C for 1 h. Then, 2 mL of 3,5-dinitrosalicylic acid (DNS) reagent was added, and the mixture was boiled for 5 min. The absorbance was determined at 540 nm. One unit of CMCA and FPA was defined as the amount of enzyme releasing 1 mol of reducing sugars per minute. All enzyme assays were performed in triplicate.

The cellulase-producing strains were molecularly characterized through 16S rRNA gene sequencing [23]. Genomic DNA was isolated with a TIANamp bacterial DNA kit (Tiangen, Beijing, China). The 16S bacterial rRNA gene sequence was amplified by 27F with sequence 5′-AGAGTTTGATCATGGCTCAG-3′ and by 1492R with sequence 5′-CTACGGTTACCTTGTTACGAC-3′. The gene sequence of 16S rRNA was verified by Sanger sequencing, which was sequenced by Sangon Biotech, Shanghai, China. A phylogenetic tree was constructed using neighbor-joining and maximum-likelihood methods with MEGA 7 software, and bootstrap values were calculated from 1000 replications.

### 2.6. Effects of Cellulase-Producing Strains on BSFL Digesting Corn Straw

Four cellulase-producing strains—designated as L1, L2, L3, and L4—isolated from BSFL guts were employed. The four strains were cultured in lysogeny broth medium (10 g/L tryptone, 5 g/L yeast extract, and 10 g/L NaCl) at 30 °C with shaking at 160 rpm, and the growth curves were monitored. Then, the cells of the log phase were centrifuged at 5000× *g* for 10 min to harvest the pellets, and the pellets were resuspended with lysogeny broth medium. The number of cells was calculated by a hemocytometer. The 10^10^ log phase cells of the four stains were added into 15 g of CS and completely soaked with water. The CS without any extra bacteria but equal lysogeny broth medium was set as the control (CK). A total of 100 five-day-old larvae were selected and reared in the plastic box. All the measurements were carried out in triplicate. The rearing boxes of BSFL were placed in a biochemical incubator at 28 °C and 70% humidity, as described in 2.2 of Materials and Methods. The growth of the BSFL was monitored every 2 days. After rearing, the analysis of CS was the same as in 2.3 of Materials and Methods.

### 2.7. Statistical Analysis

Three different substrates, namely SD, CS, and PCS, were applied to rear BSFL. Each substrate was set at three replicates, offering a total of 9 rearing boxes for the analysis of the growth of BSFL, digestion of corn straw, and gut microorganisms. Additionally, four cellulose-producing strains, designated as L1, L2, L3, and L4, were inoculated into the CS-rearing boxes, with each strain and CK group replicated three times, offering a total of 15 rearing boxes.

The weight of BSFL, survival rate of BSFL, and residual weight of the substrates were presented as means ± standard deviation (SD) (n = 3). ANOVA, followed by Tukey’s post hoc test, was used to calculate the characterization differences in all groups. GraphPad Prism software 9.0 (GraphPad Software, San Diego, CA, USA) and Microsoft were applied for diagrams construction.

## 3. Results

### 3.1. Growth of BSFL

**Figure 1:**
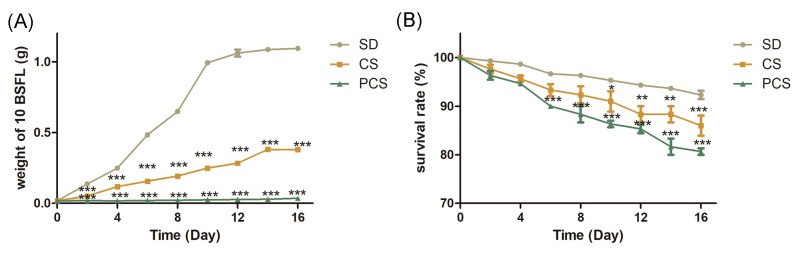
Growth of BSFL. (**A**) Weight curves of the 10 BSFL in SD, CS, and PCS. (**B**) Survival rates of 100 BSFL in SD, CS, and PCS. Values are the mean of three biological replicates. Error bars are the standard deviations from these replicates. ANOVA was used in evaluating statistical significance, and the data were analyzed and compared with those in SD. *, *p*< 0.05; **, *p* < 0.01; ***, *p* < 0.001.

A total of 100 five-day-old larvae were selected and transferred to SD, CS, and PCS. The transfer time was labeled as day 0, and the weights of 10 randomly selected BSFL were recorded every 2 days and placed back in the plastic box. Residual CS and larvae were separated before the BSFL began to transform into prepupae. As shown in Figure 1A, the weight of the BSFL in SD peaked at 1.06 g on the 12th day, and the maximum weight of the BSFL in CS was 0.39 g on the 14th day. However, the BSFL in PCS showed almost no growth (Figure S1). After the BSFL were reared for 16 days, the survival rates of the SD, CS, and PCS feedstocks were 92%, 88%, and 81%, respectively (Figure 1B). Although the standard substrate was optimal for growth, BSFL were able to survive with CS as the sole substrate, which could supply BSFL with nutrients and guarantee the survival rate. Contrary to expectations, PCS did not facilitate its digestion by BSFL; instead, it negatively impacted both the growth and survival rates.

### 3.2. Digestion of Corn Straw

**Figure 2:**
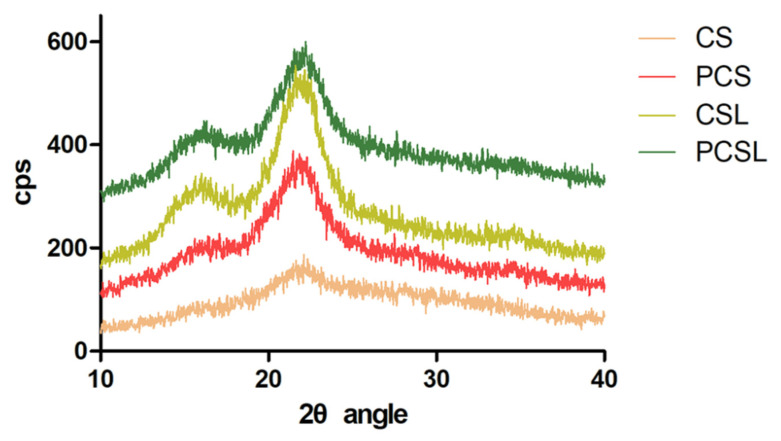
Determination of the crystallinity of corn straw by XRD.

**Figure 3:**
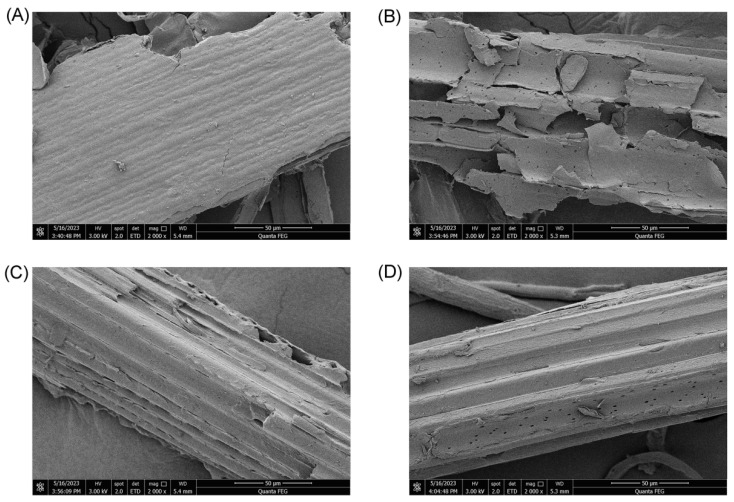
Surface morphology of corn straw observed by SEM: (**A**) CS; (**B**) CSL; (**C**) PCS; and (**D**) PCSL.

The digested residues were separated from the BSFL and freeze-dried before the BSFL were about to stop feeding and become pre-pupae. The weights of the residues were recorded. The BSFL were able to utilize almost all of the standard substrates. The BSFL could digest 37.4% and 12.0% of CS and PCS, respectively (Table 1). Thus, alkaline hydrogen peroxide pretreatment hindered the ability of the BSFL to degrade corn straw. The crystallinity of corn straw was measured by XRD. As shown in Figure 2, the crystallinities of CS and PCS were calculated to be 29.14% and 53.3%, respectively. The crystallinity of CSL increased to 61.32% after rearing with BSFL. This result suggested that the BSFL mainly digested the amorphous region, which was easy to degrade. The crystallinity of PCSL was 52.87%, which was similar to that of PCS, verifying that the BSFL almost did not grow in PCS. The surface morphology of corn straw was observed by SEM. As shown in Figure 3, the surface of CS was smooth, whereas PCS had furrows, which might be because lignin and hemicellulose were partially removed after alkaline hydrogen peroxide pretreatment. After the BSFL digested CS, the texture of CSL was unclear, and there was obvious erosion that was possibly caused by digestion. The surface of PCSL almost did not change, indicating that the BSFL did not digest PCS.

### 3.3. Larval Gut Microorganisms and the Prediction of Microbial Function

**Figure 4:**
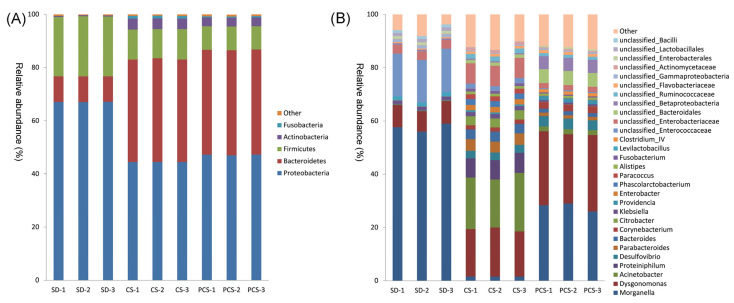
Bacterial community compositions of BSFL at the phylum and genus levels. (**A**) Top 6 ASVs (phyla). (**B**) Top 30 ASVs (genera).

Sequencing data revealed that the bacterial community structure considerably changed at the phylum level in the CS and PCS groups, compared with that in the SD group. As shown in Figure 4A, for SD, the relative abundances of Firmicutes and Proteobacteria were 22% and 67%, respectively. The abundant phyla in CS were Proteobacteria (44%), Bacteroidetes (38%), and Firmicutes (11%). The abundant phyla in PCS were Proteobacteria (47%), Bacteroidetes (39%), and Firmicutes (9%). The abundant genera in SD were *Morganella* (58%), *unclassified_Enterococcaceae* (16%), and *Dysgonomonas* (8%). However, the abundant genera in CS were *Acinetobacter* (20%), *Dysgonomonas* (18%), and unclassified *Enterobacteriaceae* (8%). The abundant genera in PCS were *Morganella* (28%), *Dysgonomonas* (27%), and *unclassified_Bacteroidales* (5%). The dominant genera within these three groups showed notable variation, indicating that the structure and composition of the intestinal microbial community of the BSFL were influenced by the type of feedstock. Interestingly, although the phyla in CS and PCS were similar, significant differences in the genera were observed (Figure 4 and Figure S2), which might be attributed to the effects of alkaline hydrogen peroxide pretreatment.

**Figure 5:**
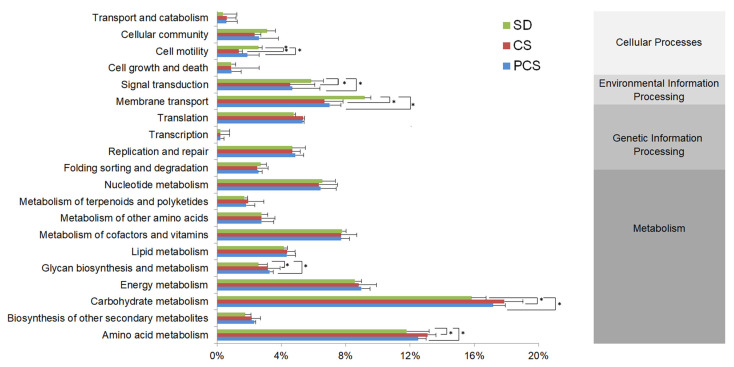
Predicted functions of the gut bacterial community at KEGG pathway level 2 analyzed by PICRUSt, based on the KEGG database. ANOVA was used in evaluating the statistical significance, and the data were analyzed and compared with those in SD. * *p*< 0.05; ** *p* < 0.01.

Through KEGG pathways (Figure 5), four primary functional gene groups, namely metabolism, genetic information processing, cellular processes, and environmental information processing, were found in all samples. The relative abundances of metabolism in SD, CS, and PCS were 65.5%, 70.9%, and 69.2%, respectively. In particular, carbohydrate metabolism had the highest relative abundance with CS as the substrate, suggesting that the substrates induced the functional genes to supply the carbon source for gut microorganisms. Compared with the standard substrate, the relative abundances of amino acid metabolism and glycan biosynthesis and metabolism were also slightly higher in CS and PCS. However, CS and PCS had lower relative abundances in signal transduction, membrane transport, and cell motility, indicating that the gut microorganisms reduced environmental information processing and the cellular process to economize energy. The bioconversion and biodegradation of corn straw were primarily attributed to biological enzymes secreted by gut microorganisms. As shown in Figure S3, the gene abundances of β-glucosidase and α-glucosidase, which were the key enzymes in lignocellulose degradation, were higher in CS and PCS than in SD. This result suggested that the gut microbial community of the BSFL was able to adjust the suitable and stable microflora in response to changes in the environment.

### 3.4. Cellulase-Producing Bacteria Assisting BSFL in Digesting Corn Straw

Four cellulase-producing strains, L1, L2, L3, and L4, were isolated from the BSFL guts. L1 was identified as *Klebsiella pneumoniae*, L2 was *Proteus mirabilis*, L3 was *Klebsiella oxytoca*, and L4 was *Providencia rettgeri* (Figures S4 and S5). Among these four strains, *K. pneumoniae* showed the best cellulose-producing ability (Table S2). Four corn straw substrates were supplemented with 10^10^ cells of the four stains, respectively, and the BSFL were reared in these boxes. As shown in Table 2, with the addition of cellulase-producing strains, the conversion rates of CS increased, compared with the control group, suggesting that cellulase-producing bacteria assisted the BSFL in digesting corn straw. The weights of the BSFL increased by 12.9%, 6.7%, 10.5%, and 3.0%, and the survival rates increased by 10%, 6.5%, 7.1%, and 5.3%, respectively. These results indicated that cellulase-producing bacteria were beneficial to the growth of the BSFL. In particular, *K. pneumonia* and *K. oxytoca* showed the best abilities in increasing the conversion rate of corn straw and the weight of the BSFL. *K. pneumonia* displayed the best benefits to the survival rate, which were positively correlated with the cellulase-producing abilities. These results might provide guidance for improving the cultivation of BSFL using corn straw.

## 4. Discussion

Crop straw is considered a renewable biomass energy source. In China, the annual corn straw yield amounts to 350 million tons [2]. An effective method for using straw is necessary. In recent years, the utilization of insects for the treatment of crop straw has become a topic worthy of attention [24]. BSFL are saprophytic insects recognized for their efficiency in converting organic waste [25,26]. Some studies have attempted to rear BSFL with rice straw, but the conversion rates were not outstanding. Liu et al. reported that the alkaline peroxide pretreatment of rice straw is beneficial for enhancing the conversion rate [11]. In this study, we found that the effective conversion rate of corn straw to BSFL was 37.4% (Table 1), which was more efficient than the previously reported rate of 10.85% for rice straw [9,10]. Moreover, BSFL development in corn straw was significantly faster, taking approximately 20 days, compared to 39 days in rice straw. These results suggested that corn straw was more suitable as the substrate of BSFL than rice straw. To increase the conversion rate, we applied alkaline peroxide pretreatment to corn straw; however, the results demonstrated that this pretreatment was detrimental to both the conversion efficiency of corn straw and the growth of BSFL. This result might be due to the residues of sodium hydroxide and hydrogen peroxide. Studies showed that alkaline peroxide pretreatment also removed the sugar, fat, starch, and protein from corn straw, which might affect the growth of BSFL [11,27]. In addition, the pretreatment of straw with alkaline hydrogen peroxide to improve the conversion rate of BSFL is not an environmentally friendly method. Bio-friendly ways to improve the utilization of straw and the growth of BSFL are necessary, such as the pretreatment of corn straw with lignocellulose-degrading strains and spontaneous fermentation to disturb the compact structure of lignocellulose. Notably, the pretreatment of corn straw influenced the community structure of the gut microorganisms.

During the degradation of organic material, BSFL initially masticated straw using their mouthparts, followed by the passage of the resulting fragments into the intestine [28]. However, BSFL growth on CS as the sole substrate was inferior to that on SD. After 16 days of rearing, the average weight of a single larva on SD was 106 mg, whereas it was 39 mg on CS (Figure 1). These results suggested that compared with SD, CS was unsuitable for rearing BSFL, but most of the BSFL did grow and survive in this substrate, indicating that all the nutrients needed for BSFL growth could be provided by CS. However, the limited biomass of BSFL in CS presents a challenge for its immediate industrial-scale use in rearing BSFL. The chemical composition of the BSFL indicated that the total of crude protein and ether extract of BSFL in CS was 8% less than that of SD (Table S1), suggesting that the CS was an alternative to rearing BSFL and obtaining crude protein and ether extract. The study provided guidance for rearing BSFL with CS. Finding an optimal ratio of CS and SD and the proper pretreated methods of CS would be worthwhile research directions towards using CS as a substrate to rear BSFL.

By analyzing the gut microorganisms, we found the corn straw-shaped gut microorganisms’ diversity and revealed the corn straw-dependent characteristics of gut microorganisms. Compared with the SD, the relative abundance of Bacteroidetes in CS increased by 28%, suggesting the importance of Bacteroidetes in digesting lignocellulose (Figure 4). Meanwhile, based on the KEGG prediction, the gene abundances of some lignocellulose degradation enzymes, such as β-glucosidase and α-glucosidase, were higher in CS than in SD (Figure S3), indicating the importance of gut microorganisms in the degradation of lignocellulose. According to the predicted functions of the gut bacterial community at KEGG pathway level 2 (Figure 5), cell motility and signal transduction were lower than that of the SD group, indicating that the gut bacterial community reduced cell activity to accumulate energy to digest CS. The abundance of *Morganella* in the SD group was 58%, while that of the CS group was only 1.5%. In addition, researchers showed that *Morganella* contained many genes for cell signaling and motility [29], which might be caused by the reduction of cell motility and signal transduction in the CS group. Compared with the SD group, the abundance of *Proteiniphilum* increased by 7.2% in the CS group. *Proteiniphilum* was able to cooperate with lignocellulose-degrading strains to convert CS into acetic, propionic, and isovaleric acids for the growth of BSFL [30]. There was almost no *Proteiniphilum* tested in the PCS group, which might be a resulted of the barely growing BSFL in PCS. Therefore, the changes in the gut bacterial community suggested that the larval gut bacterial community was able to adapt to the substrate of CS and possessed enough ability to digest CS.

The pure culture technique was adopted to isolate cellulase-producing strains from the gut microbiota of BSFL. Unfortunately, the cellulase-producing strains from Bacteroidetes were not isolated from the BSFL gut, possibly due to the adoption of aerobic nutrition screening methods [31]. Diversity screening methods could be applied to screen additional functional strains. Four Proteobacteria cellulase-producing strains from the BSFL guts were found to be beneficial to the conversion rate of CS, growth of BSFL, and survival rate. The strains were helpful in degrading lignocellulose and thus assisted the growth of BSFL. Above all, the benefit was related to the cellulase-producing ability. That is, we might apply other safe cellulase-producing strains to increase the conversion rate of corn straw, growth of BSFL, and survival rate in the future, considering the pathogenicity of *K. pneumonia*. The addition of cellulase-producing strains to the system might provide a bio-friendly strategy to optimize the rearing of BSFL on CS.

## 5. Conclusions

In summary, this study reported that BSFL can survive and gain weight with CS as the sole feedstock. The bacterial community structure of the CS group was significantly different from that of the SD group. The response mechanism of gut microorganisms to CS stress was studied. Four cellulose-producing strains from the BSFL gut were identified, which were beneficial to the growth of BSFL. This report presented guidance for rearing BSFL with CS. This study about promoting the accumulation of the BSFL biomass in CS will be worthwhile.

## Figures and Tables

**Table 1:** Developments of BSFL with three substrates.

Sample	Weight of Initial Substrates (g)	Residual Weight of Substrates (g)	Conversion Rate (%)
SD	15	0.51 ± 0.01	96.6 ± 0.06
CS	15	9.45 ± 0.22 ***	37.4 ± 1.47 ***
PCS	15	13.20 ± 0.18 ***	12.0 ± 1.21 ***

Note: The residual weights of substrates were the averages of three triplicates ± standard deviation. The data are expressed as the mean ± standard deviation. *** indicates significant differences (*p* < 0.001).

**Table 2:** Effects of different bacteria on the weights of BSFL.

Sample	Residual Weight of Substrates (g)	Conversion Rate (%)	Weights of 10 BSFL (g)	Survival Rate (%)
CK	9.45 ± 0.22 ^a^	37.4 ± 1.47 ^a^	0.372 ± 0.012 ^a^	85.0 ± 0.5 ^a^
L1	8.36 ± 0.21 ^b^	44.3 ± 1.40 ^b^	0.420 ± 0.023 ^b^	93.5 ± 1.0 ^b^
L2	8.74 ± 0.11 ^c^	41.7 ± 0.73 ^c^	0.397 ± 0.011 ^c^	90.5 ± 0.5 ^cd^
L3	8.48 ± 0.17 ^b^	43.5 ± 1.13 ^b^	0.411 ± 0.013 ^b^	91.0 ± 0.5 ^c^
L4	8.75 ± 0.21 ^c^	40.7 ± 0.40 ^c^	0.357 ± 0.012 ^d^	89.5 ± 0.5 ^d^

Note: Residual weights of substrates, weights of 10 BSFL, and survival rate were the averages of three triplicates ± standard deviation. Different lower-case letters indicate significant difference (*p* < 0.05).

## Data Availability

The original contributions presented in the study are included in the article/Supplementary Material, further inquiries can be directed to the corresponding author.

## Supplementary Materials

The following supporting information can be downloaded at: https://www.mdpi.com/article/10.3390/insects15100734/s1. Figure S1: The appearances of BSFL reared by three substrates at day 0 and day 16; Figure S2: Principal component analysis (PCA) analysis of gut DNA samples based on genus.; Figure S3: Heatmap of KEGG functional prediction; Figure S4: The colony forms and individual forms of the four cellulose-producing strains; Figure S5: The phylogenetic trees of the four cellulose-producing strains; Table S1: The composition of BSFL reared on different substrates; Table S2: Cellulase-producing ability of four cellulase-producing strains.
