# Supplementary material for: Rearing of Black Soldier Fly Larvae with Corn Straw and the Assistance of Gut Microorganisms in Digesting Corn Straw
Source: Insects. 2024 Sep 24;15(10):734. doi: 10.3390/insects15100734 (PMC11508837; doi:10.3390/insects15100734)
Supplement: Supplementary file 1 [file insects-15-00734-s001.zip › insects-3145693-supplementary.pdf]

Title

Rearing of Black Soldier Fly Larvae with Corn Straw and the Assistance of Gut Microorganisms in Digesting Corn Straw

Authors

Xifeng Wang[1*], Xiangru Tian[1], Zhi Liu[1], Zhihua Liu[1], Shuying Shang[1,2], Haifeng Li[1], Jianhang Qu[1], Pengxiao Chen[3]

Authors' affiliation

1. School of Biological Engineering, Henan University of Technology, Zhengzhou 450001, China

2. School of International Education, Henan University of Technology, Zhengzhou 450001, China

3. School of Food and Strategic Reserves, Henan University of Technology, Zhengzhou 450001, China

* Corresponding Author Address: College of Biological Engineering, Henan University of Technology, Zhengzhou 450001, China

Tel.: +86-371-67756533.

E-mail: wangxifeng@haut.edu.cn

Supplemental Materials

Table S1 The composition of BSFL reared on different substrates.

Substrates	Crude Protein (%)	Ether extract (%)	Ash (%)
SD	36.62 ±0.35	31.80 ± 0.32	5.73 ±0.22
CS	41.73 ±0.65*	18.75 ± 0.23**	13.67± 0.34**

The data is expressed as the mean ± standard deviation. Significant differences (T-test *P < 0.05, **P < 0.01) were detected between SD and CS.

Table S2 Cellulase-producing ability of four cellulase-producing strains.

Strains	transparent zone (mm)	Enzyme activity of CMC (U/mL)	Enzyme activity of FPA (U/mL)
L1	5.7 ± 0.2^{a}	48.01 ± 0.18^{a}	45.81 ± 0.32^{a}
L2	5.1 ± 0.1^{b}	38.46 ± 0.22^{b}	36.50 ± 0.13^{b}
L3	5.5 ± 0.1^{a}	40.08 ± 0.21^{c}	42.65 ± 0.23^{a}
L4	4.7 ± 0.1^{c}	36.52 ± 0.12^{bc}	28.66 ± 0.14^{c}

The data is expressed as the mean ± standard deviation. Different lower-case letters indicate significant difference (P < 0.05).

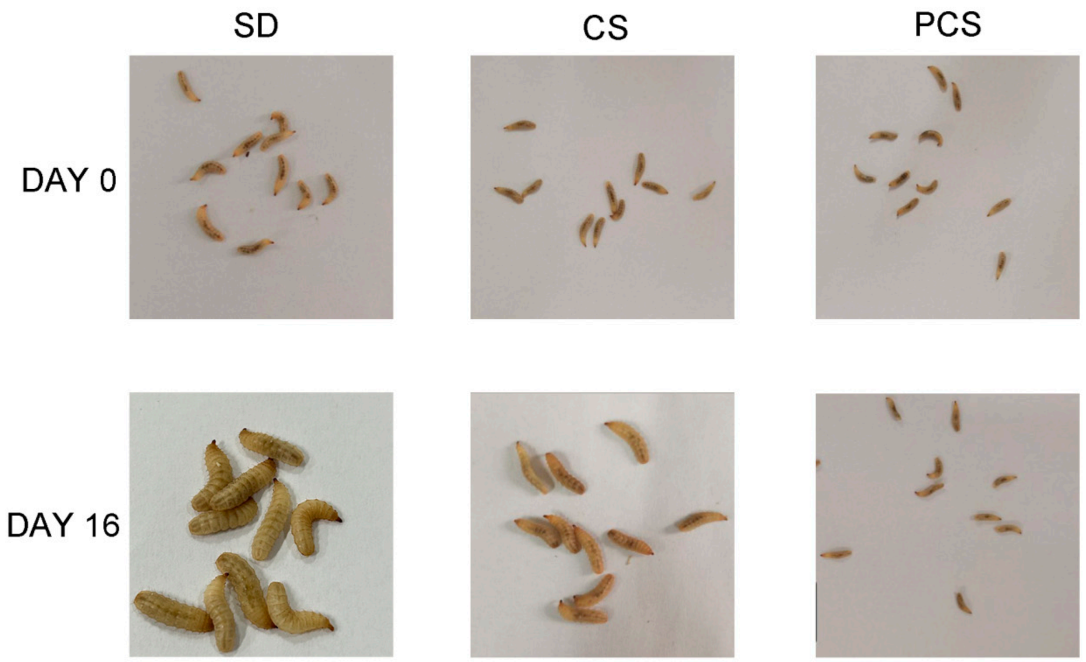

Figure S1 The appearances of BSFL reared by three substrates at day 0 and day 16.

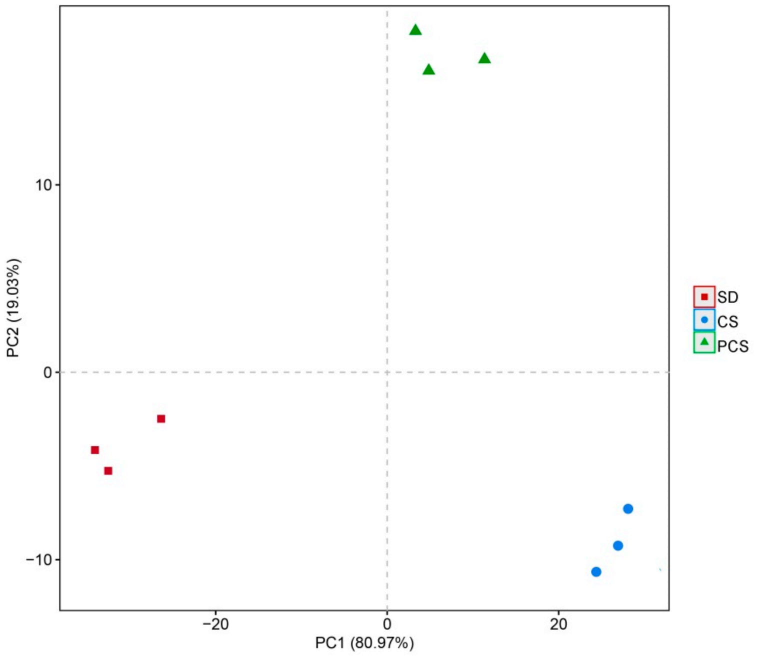

Figure S2 Principal component analysis (PCA) analysis of gut DNA samples based on genus.

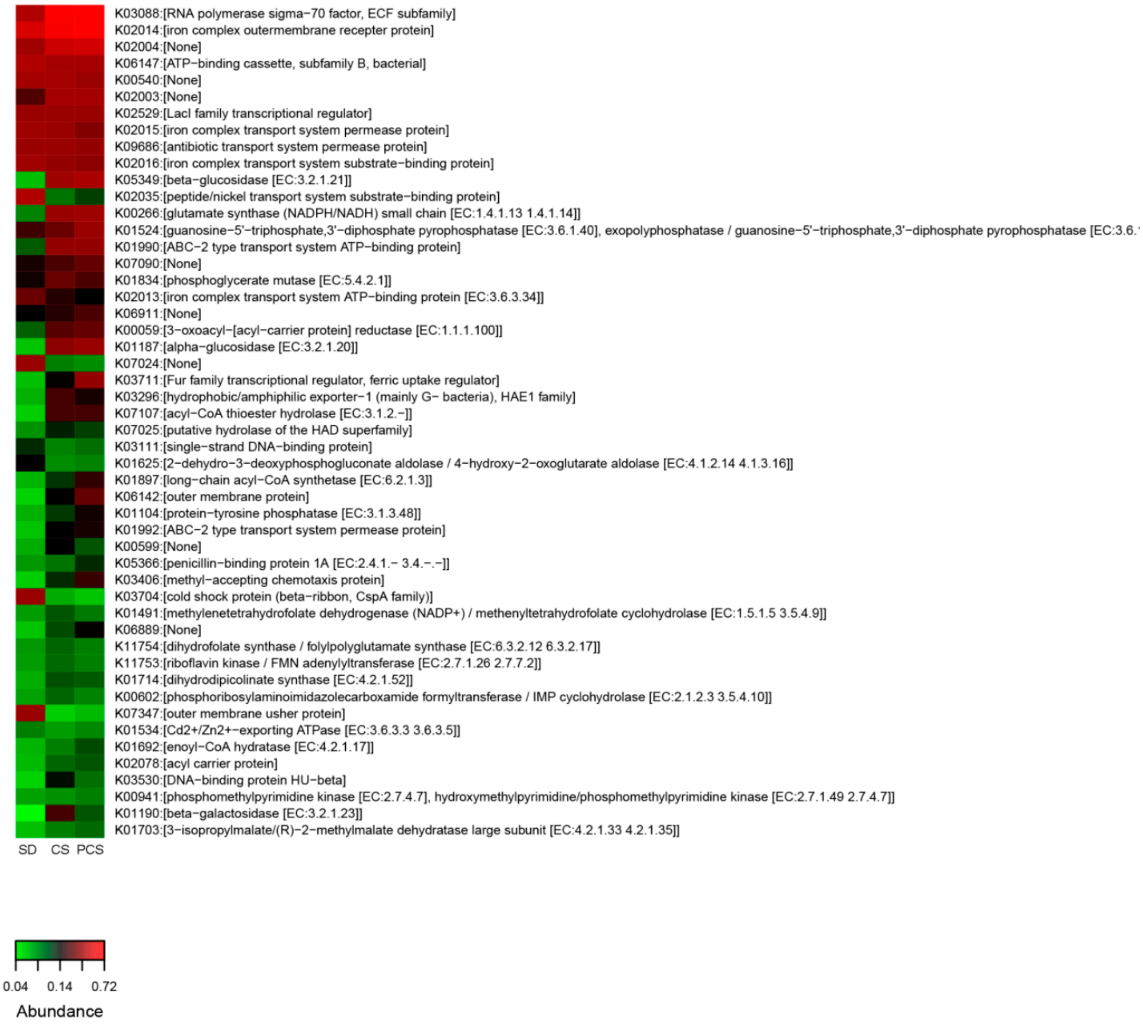

Figure S3 Heatmap of KEGG functional prediction.

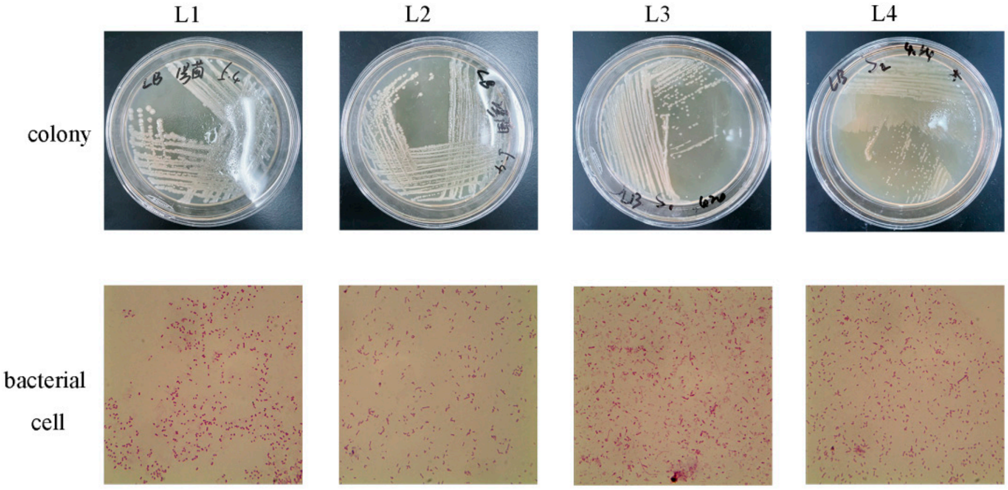

Figure S4 The colony forms and individual forms of the four cellulase-producing strains.

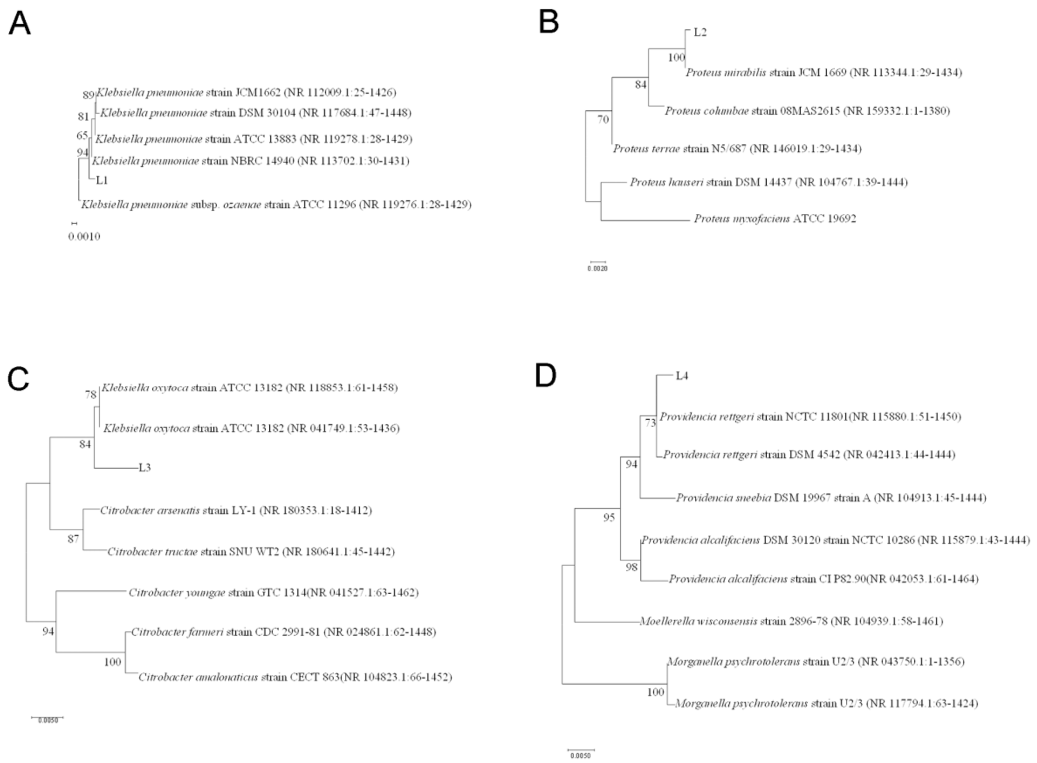

Figure S5 The phylogenetic trees of the four cellulase-producing strains.
